# Computational and technical approaches to improve the implementation of prevention programs

**DOI:** 10.1186/1748-5908-10-S1-A28

**Published:** 2015-08-20

**Authors:** C Hendricks Brown, Craig PoVey, Arthur Hjorth, Carlos G Gallo, Uri Wilensky, Juan Villamar

**Affiliations:** 1 Departments of Psychiatry and Behavioral Sciences, Preventive Medicine, and Medical Social Sciences, Fienberg School of Medicine, Northwestern University, Chicago, IL 60611, USA; 2 Utah Department of Substance Abuse and Mental Health, Salt Lake City, UT 84116, USA; 3 Center for Connected Learning & Computer-Based Modeling, Northwestern University, Evanston, IL 60208, USA

## Introduction

A potential new arena that could lead to major advances in implementation science involves the integration of computational and technologic approaches with behavioral and organizational sciences. While some attention has been given to the use of systems science methods, specifically agent-based modeling, social network analysis, and system dynamics, there is actually a much broader set of tools that could be used to improve adoption, assessment of fidelity, and sustainability. This panel provides a broad perspective and illustrates how such tools can aid implementation especially given the unique challenges in the prevention field.

## Methods

In Brown et al (2013) we proposed (1) the use of advance computational approaches to address implementation of effective prevention programs and (2) a general systems model to support the implementation of prevention programs. Regarding computational approaches, an automated fidelity system that uses computational linguistics is shown to provide reliable and cost-effective means for monitoring fidelity of a behavioral intervention. Also, a machine learning algorithm is used to monitor text and discover when organizations are or are not making consistent progress in implementation. Agent-based modeling using Netlogo is used to inform policy makers and community leaders regarding the impact they are likely to see in their communities. In terms of technology, a mobile phone app is used as a pilot for teachers, that is implementation agents, to improve the fidelity of delivering a prevention program.

## Results

Computational approaches provide a platform in which to design and test tools that aid the implementation and dissemination of evidenced-based prevention programs.

## Discussion

We discuss how these automation tools can be used to increase health equity as well as provide more cost-effective and supportive feedback systems for implementation.

Computational approaches to implementing effective programs to prevent HIV in minority communities

African Americans and Hispanics in the United States have much higher rates of HIV than non-minorities. There is now strong evidence that a range of behavioral interventions are efficacious in reducing sexual risk behavior in these populations. Although a handful of these programs are just beginning to be disseminated widely, we still have not implemented effective programs to a level that would reduce the population incidence of HIV for minorities.

We proposed that innovative approaches involving computational technologies be explored for their use in both developing new interventions and in supporting wide-scale implementation of effective behavioral interventions. Mobile technologies have a place in both of these activities. First, mobile technologies can be used in sensing contexts and interacting to the unique preferences and needs of individuals at times where intervention to reduce risk would be most impactful. Second, mobile technologies can be used to improve the delivery of interventions by facilitators and their agencies. Systems science methods including social network analysis, agent-based models, computational linguistics, intelligent data analysis, and systems and software engineering all have strategic roles that can bring about advances in HIV prevention in minority communities.

We first illustrate how 8 areas in the implementation process can use innovative computational approaches to advance intervention adoption, fidelity, and sustainability. We next discuss how a mobile app can improve implementation of the Good Behavior Game, a behavioral intervention delivered by teachers in first-grade classes. Thirdly we describe how machine learning can automate assessment of the implementation stages of the Familias Unidas intervention, which is delivered to families of Hispanic adolescents.

These methodologies have promise in overcoming the large number barriers in addressing implementation challenges especially for prevention.

Using agent-based modeling to visualize the effects of prevention implementation strategies for policy

In contrast to treatment, the effects of behavioral prevention programs are challenging to see, as it is the absence of problem behavior, diagnoses, and disorders that characterize successful programs. Lessening such outcomes, often occurring over years and never completely extinguished, are not at all evident for policy makers or community leaders. In considering whether to implement a prevention program, community leaders would like to visualize how outcomes would be affected by such a program and compare projected outcomes in real-time.

In response to this need, we have developed a NetLogo agent-based model as a general visualization tool to compare outcomes with, as well as without, implementing such an intervention. Agent-based modeling is a computer-based simulation approach in which the modelers specify behaviors at the agent-level and explore the system-level outcomes that emerge. Its focus on interactions among heterogeneous individuals makes it particularly well-suited for studying implementation of behavioral interventions. The model can be customized to the risk and protective factors of the existing community, developmental trajectories of subjects with and without exposure to an intervention, much like viewing two movies side by side with different endings.

This side-by-side comparison uses input from longitudinal studies, preventive and implementation trials to simulate short and longer-term outcomes. We illustrate this approach by examining the effects of the Good Behavior Game, a prevention program tested in first grade that reduces aggressive disruptive behavior as well as long-term sequelae of drug abuse/dependence disorder, conduct disorder, juvenile and adult arrests, and suicidal ideation and attempts. We highlight how interactions among highly aggressive youth in classes where aggression is normative versus non-normative lead to differential effects. Implications for when implementation of this program is likely to have maximal impact are given.

Using computational linguistics to overcome implementation barriers in fidelity assessments of a behavioral intervention

Careful fidelity monitoring and feedback are critical to implementing effective interventions. Most procedures to assess fidelity are derived from observational assessments. However, these fidelity assessments are resource intensive for research teams in efficacy/effectiveness trials, and often unattainable or unmanageable for host organizations. Thus, it is difficult to evaluate when the intervention is implemented on a large scale. Our goals are to 1) operationalize fidelity using a rule based system, and 2) develop tools that use these rules to assess fidelity automatically.

Our data consists of 40 videotaped sessions from the effectiveness trial of the Familias Unidas, an evidence-based, family-centered preventive intervention found to be efficacious in reducing conduct problems, substance use, and HIV sexual risk behaviors among Hispanic youth. We use an innovative mixed methods approach to 1) uncover linguistic patterns linked to "joining," which measures the quality of the working alliance of the facilitator with the family, 2) to use computational linguistics to reduce human effort and provide real-time fidelity assessments to supervisors. Quantitative assessments of reliability are provided. Kappa scores between a human rater and a computer rater for the new method for measuring joining reached 0.83.

Early findings suggest that this approach can reduce 1) the high cost of fidelity measurement, 2) the time delay between session delivery and fidelity feedback to facilitators, 3) the number of sessions (~90%) for which fidelity is not assessed. This approach also has the potential for informing parent training intervention theory about effective language to engage participants.

